# Hederagenin Attenuates Cerebral Ischaemia/Reperfusion Injury by Regulating MLK3 Signalling

**DOI:** 10.3389/fphar.2020.01173

**Published:** 2020-07-30

**Authors:** Hailong Yu, Lilong Song, Xiang Cao, Wei Li, Yuanyuan Zhao, Jian Chen, Jun Li, Yingzhu Chen, Wenkui Yu, Yun Xu

**Affiliations:** ^1^Affiliated of Drum Tower Hospital, Medical School of Nanjing University, Nanjing, China; ^2^Clinical Medical College of Yangzhou University, Yangzhou, China; ^3^Department of Neurology, Northern Jiangsu People’s Hospital, Yangzhou, China; ^4^Dalian Medical University, Dalian, China

**Keywords:** cerebral ischaemia/reperfusion injury, hederagenin, apoptosis, inflammation, MLK3 signalling pathway

## Abstract

Cerebral ischaemia/reperfusion (CI/R) injury is a major challenge due to the lack of effective neuroprotective drugs. Hederagenin (HE) is the aglycone part of saponins extracted from *Hedera helix Linné* that has exhibited anti-apoptotic and anti-inflammatory effects; however, the role of HE in CI/R has not been elucidated. In this study, mice were intraperitoneally (i.p.) injected with HE (26.5, 53, or 106 μmol/kg body weight) for 3 days after middle cerebral artery occlusion (MCAO). Neural function and brain infarct volume were evaluated. HE treatment attenuated CI/R-induced apoptosis and inflammatory cytokine expression within the infarcted areas. HE treatment also decreased the activation of the MLK3 signalling pathway, which potentiates CI/R damage *via* the MAPK and NFκB pathways. Due to HE’s safety profile, it has potential to be used for the clinical treatment of ischaemic stroke.

## Introduction

Ischaemic stroke is currently the leading cause of death and disability worldwide (Benjamin et al., 2017). Due to the narrow window of opportunity for administration of thrombolytic therapy, few patients meet clinical criteria for its use (Balami et al., 2013). There remains a critical need to identify safe and efficacious neuroprotective agents.

Hederagenin (HE), a triterpenoid which is the aglycone part of numerous saponins isolated from ivy (*Hedera helix Linné*) leaves, has been reported to have anti-tumour, anti-apoptosis, and anti-inflammatory effects (Zhang et al., 2012; Lu et al., 2015). Apoptosis and inflammation are both key molecular pathways involved in cerebral ischaemia/reperfusion (CI/R) injury. Previous studies have shown that HE decreases the expression of apoptotic markers and inflammatory cytokines through modulating the MAPK, NFκB, and PI3K/AKT pathways (Lu et al., 2015; Kim et al., 2017) in other disease models. Recent research has demonstrated that HE can pass through the blood-brain barrier (BBB) (Yang et al., 2011; Liang et al., 2015). In the treatment of depression and mood disorders, HE was found to inhibit serotonin secretion (Liang et al., 2015); however, the effect of HE on CI/R injury is still unclear. According to the biological characteristics of HE, we speculated that HE may exert protective effects against CI/R injury.

In this study, we explored the pharmacological roles and molecular mechanism of HE on CI/R injury using a mouse model of middle cerebral artery occlusion (MCAO).

## Materials and Methods

### Materials

HE (Nanjing Spring & Autumn Biological Engineering Co. Ltd) with a purity greater than 98% was dissolved in 2% dimethyl sulfoxide (DMSO) to generate stock solutions. 2,3,5- triphenyltetrazolium chloride (TTC) was purchased from Sigma-Aldrich; rabbit beta-actin polyclonal antibody, rabbit anti-Bcl-2, and rabbit anti-Bax were purchased from Bioworld Technology Inc. (St. Louis Park, MN, USA). Rabbit beta-IKK polyclonal antibody were purchased from Cell Signalling Technology; RIPA lysis buffer was purchased from Millipore (Billerica, MA, USA). A BCA Protein Assay Kit was purchased from Thermo-Fisher Scientific (MA, USA); anti-beta-tubulin, anti-NF-κB (p-p65), anti-SAPK/JNK, anti-phospho-SAPK/JNK, and goat anti-rabbit secondary antibody were purchased from Cell Signaling Technology. Goat anti-Iba-1 was purchased from Abcam (1:500); Trizol was purchased from Invitrogen (USA). A PrimeScript RT reagent kit was purchased from Takara (Dalian, China); Lv-MLK3 and Lv-con were purchased from GeneChem (Shanghai China). A SYBR green Kit was purchased from Applied Biosystems (Foster City, CA, USA) and the ECL chemiluminescence system was purchased from Thermo Company (Rockford, IL, USA).

### Animals

Seven-to-eight-week-old male C57BL/6J (B6) mice (Nanjing University Animal Model Centre) were used in these experiments. Mice had an average weight of 20‑25 g. The mice were housed in standard plastic cages, fed a constant diet of solid food, provided water ad libitum, and maintained in 12-h light-dark cycles under relative humidity of 55 ± 5% at 22 ± 2°C (Cheng et al., 2019). All experimental procedures were approved by the Animal Ethics Committee of Nanjing University.

### Grouping and Drug Administration

**Figure 1:**
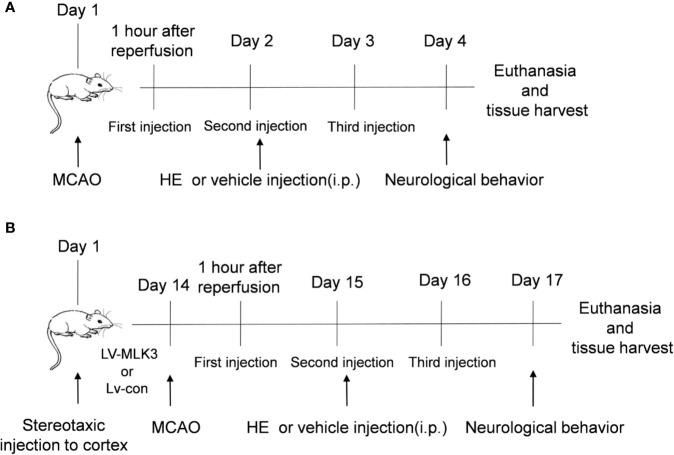
Schematic representation of the study design. **(A)** The time point of the first HE treatment was 60 min after MCAO. HE was then injected once daily for 3 consecutive days. After 3 days of MCAO, the mice were sacrificed following completion of the neurological behaviour assessments. The brain tissue was the harvested for subsequent research. **(B)** In this study, lentiviruses were used to transfect the cortex of the mouse brains. The mice then underwent MCAO 14 days after transfection. Subsequent research was conducted as described in part A.

Mice were randomized into four groups as follows: I) control vehicle-treated group (Sham group, n=25); II) HE-treated group (HE group; n= 25); III) vehicle-treated CI/R group (CI/R group; n=30); and IV) HE-treated CI/R group (CI/R + HE group; n=40). After the induction of MCAO, mice underwent intraperitoneal injections with either HE (26.5, 53, or 106 umol/kg body weight) or the vehicle (2% DMSO in normal saline) daily for 3 consecutive days (**Figure 1A**).

### The Middle Cerebral Artery Occlusion (MCAO) Model

The middle cerebral artery occlusion model was performed with mice under general anesthesia with 1.5% isoflurane in a 30% O2/68.5% N_2_O mixture. Focal cerebral ischaemia was induced by the intraluminal occlusion of the right MCA for 1 h. The right common carotid artery (CCA), the right external carotid artery (ECA), and the internal carotid artery (ICA) were exposed through a ventral midline neck incision. The ECA was ligated with a silk suture at 2 mm distal from the ECA–CCA branch and then cut distally from the ligated point. A silk suture was looped around the CCA and twisted to block blood flow from the CCA. A small incision was made on the ECA 1.5 mm distal from the ECA–CCA branch. A 6-0 nylon monofilament (Ethilon, Ethicon Inc) coated with silicon resin (Heraeus, Kulzer, Germany) was introduced through the incision into the right CCA and advanced 9–11 mm distal to the carotid bifurcation until resistance was felt for temporary occlusion of the middle cerebral artery. Reperfusion was achieved by withdrawing the suture after 1-h of MCAO to restore blood supply to the MCA territory. The silk suture looped around the CCA was removed and the neck incision was closed. The sham group underwent the same surgical procedure except that the monofilament was introduced into the external carotid artery but not advanced. Surgery was performed with mice under a warming blanket with temperature monitoring by anal thermometer to avoid hypothermia and maintain internal temperature of 36‑38°C.

Regional cerebral perfusion (RCP) was monitored using invasive Doppler blood flow monitoring. Through a craniotomy incision, the outer cortex of the cranium 1 mm posterior and 3 mm lateral to the Bregma was thinned. The fiber optic probe was attached to this test site by the fast gel water. Blood flow curves were recorded using MoorvMs-PC software interface for continuous monitoring of cerebral perfusion during the procedure. Mice that demonstrated greater than 70% reduction in cerebral blood flow were included in the study. Regional CBF was also monitored using Doppler two-dimensional laser speckle imaging techniques 15 min before and after the onset of MCAO (**Supplementary Figure 1A**). All MCAO mouse models were completed by experienced veterinary technicians. Mice in the sham operation group underwent the same operation except for the insertion of the filament into the MCA.

Mice with intracranial hemorrhage, those that did not survive past 3 days following MCAO, and those with less than 70% reduction in cerebral perfusion were excluded from the study.

### Lentivirus Stereotactic Injection

Similar to our previous experimental methods (Zhang X. et al., 2019), a lentiviral vector overexpressing either the MLK3 activator (LV-MLK3) or negative control (LV-con) was injected into the cerebral cortex with stereotactic guidance, after the mice were anaesthetized. Three injection points in the right cerebral cortex (0.3 mm front of the bregma, 0.8 mm behind the bregma, and 1.9 mm after the bregma; 3 mm lateral; and 1.8 mm deep) were used in each mouse. Injections were completed with a 10-μl microsyringe at a rate of 0.1 μl min^−1^. Two-weeks following injection, mice then underwent middle cerebral artery inclusion to induce CI/R injury (**Figure 1B** and **Supplementary Figure 1B**).

### Behavioural Analysis of Neurological Deficit Scores

Neuro-behavioural function was assessed using the modified neurological severity score (mNSS) and Longa score on day 3 following MCAO by a blinded observer. The mNSS evaluates neurological deficits through motor, reflex, sensory, and balance testing. The score ranges from 0, indicating no neurologic deficit, to 18 for animals with the most severe impairment. The Longa score is determined as follows: 0 point, no neurological deficit; 1 point, cannot fully extend the contralateral forelimb; 2 points, tail-catching phenomenon while walking (circling to the contralateral side); 3 points, unsteady in the standing position, falling to the contralateral side; and 4 points, no spontaneous walking and decreased consciousness. Scores of 0‑2 are classified as mild neurological impairment, while scores of 3‑4 are classified as severe neurological impairment.

The evaluators were blinded to the treatment group of mice during the neurobehavioral tests.

### Cerebral Infarction Volume Measurement

After neurological evaluation, mice were sacrificed and histological evaluation of brain tissue was performed. The brain was sectioned into six slices and incubated with 0.2% TTC at 37°C for 15 min to determine the infarct volume. Infarcted tissue was pale grey in color, compared to the dark red color of normal brain tissue. Images were obtained using a digital camera and analyzed using ImageJ software. The percentage of hemispheric infarction volume was calculated using the following formula: Infarct size = (contralateral area—ipsilateral non-infarct area)/contralateral area x 100%.

### Western Blot Analysis

Western blot analysis was then performed on the cerebral tissue within the ischemic areas. Proteins were homogenized in RIPA lysis buffer (Millipore, Billerica, MA, USA) containing protease inhibitors. The supernatant was assayed using a BCA Protein Assay Kit. The protein concentration was adjusted to the same concentration with protein buffer. The protein samples were then loaded, electrophoresis was performed, and the separated proteins were electrically transferred to a nitrocellulose or polyvinylidene difluoride (PVDF) membrane. The membrane was blocked in PBS containing 0.1% Tween-20 and 5% non-fat milk for 2 h at room temperature. The membrane was then incubated with the primary antibodies overnight at 4°C. After washing with PBST, the blots were incubated with HRP-conjugated secondary antibody in blocking solution for 2 h and developed with the ECL chemiluminescence system (Thermo Company, West Chester, PA, USA).

### Quantitative Real-Time-Polymerase Chain Reaction (qPCR)

Total RNA was extracted from the ischemic cerebral cortex using a commercial TRIzol kit, and RNA was then reverse-transcribed into cDNA using a PrimeScript RT reagent Kit. Quantitative measurements were performed on an ABI 7500 PCR instrument (Applied Biosystems, USA) using a SYBR green kit. Relative gene expression levels were normalized to GAPDH. The sequences of the primers used are as follows: TNF-a: (forward) TCGGTCCCAACAAGGAGGAG and (reverse) GGGTTGTCACTCGAGTTTTG; IL-1β: (forward) GCAACTGTTCCTGAACTCAACT and (reverse) ATCTTTTGGGGTCCGTCAACT; IL-6β: (forward) GGCGGATCGGATGTTGTGAT and (reverse) GGACCCCAGACAATCGGTTG; GAPDH (forward) GCCAAGGCTGTGGGCAAGGT and (reverse) TCTCCAGGCGGCACGTCAGA. IL-4: (forward) GGTCTCAACCCCCAGCTAGT and (reverse) GCCGATGATCTCTCTCAAGTGAT; and IL-10: (forward) GGTTGCCAAGCCTTATCGGA and (reverse) ACCTGCTCCACTGCCTTGCT.

### Immunofluorescence Staining of Frozen Sections

In brief, before brain tissue was removed, the mice were infused with normal saline after euthanasia and fixed with 4% paraformaldehyde. The brain tissue was quickly retrieved and then placed into 15% or 30% sucrose solution for gradient dehydration. First, the designated brain slices were incubated with 0.2% bovine serum albumin (BSA) to exclude non-specific staining). The brain tissue or cells were then permeated with 0.25% Triton X-100, washed with PBS and 2% BSA, sealed at room temperature for 2 h, and then incubated overnight with the designated primary antibody (goat anti-Iba-1, Abcam, 1:500) at 4°C. The tissue was then incubated with the secondary antibody (Thermo Fisher) at room temperature for 2 h in the dark. Finally, after three washes in PBS, the sections were stained with DAPI for 15 min for nuclear labelling. The images were captured using an Olympus X73 fluorescence microscope and a laser scanning confocal microscope. The positive cells were counted using Image-ProPlus6.0 software by a blinded observer.

### TUNEL Staining

TUNEL staining was used to detect the apoptosis of neurons in the ischaemic penumbra. As previously described (Yu et al., 2019), the frozen sections were dried at 37°C for 2 h, washed in PBS 3 times for 5 min each time, soaked in PBS containing 0.25% Triton X-100 for 15 min to permeabilize the cells, and washed with PBS 3 times. The cells were covered with 100 μl of equilibrium buffer and incubated at room temperature for 5‑10 min. 50 μl of terminal deoxynucleotidyl transferase recombinant (RTdT) incubation buffer was then added to each tissue slice, and the slices were then incubated for 60 min at 37°C in a dark humidified atmosphere. The slides were soaked in 2X SSC solution, and the reaction was terminated after 15 min. The cells were stained with DAPI for 15 min and then exposed to the quenchant. Laser scanning confocal microscopy was performed with a fluorescence microscope (Olympus X73). Positive cells were quantified *via* Image-Pro Plus6.0 by blinded observers.

### Flow Cytometry

Single-cell suspensions were prepared from the brain tissues of each group of mice on the 3^rd^ day after MCAO. Brain tissues were ground and homogenized in PBS and passed through 40-µm nylon cell strainers for cell collection. Cell suspensions were centrifuged at 2,000 rpm for 5 min, and the cell pellets were then resuspended in 5mL of 30% Percoll solution. The gradient was centrifuged at 2,000 rpm for 30 min at room temperature. Cells were then stained with fluorochrome-conjugated antibodies. All antibodies were purchased from BD Bioscience (Franklin Lakes, NJ, USA) or Biolegend (San Diego, CA, USA). The following mouse antibodies were used: CD45, CD11b, CD86, and CD206. Cell surface phenotype were performed on a FACS FORTESSA flow cytometer (BD Bioscience, Franklin Lakes, NJ, USA). The data were analyzed using Flow Jo software version 7.6.1 (Flow J, LLC, Ashland, OR, USA).

### Statistics

Relevant statistical analyses and graphing were performed using ImageJ and GraphPad Prism 7.0. All data are expressed as the mean ± SEM. One-way analysis of variance (ANOVA) was used for multiple comparisons among groups, and Student’s t-test was used for comparisons between two groups. P <0.05 was considered significant.

## Results

### HE Reduced Infarct Volumes and Improved Neurological Deficits After CI/R Injury

**Figure 2:**
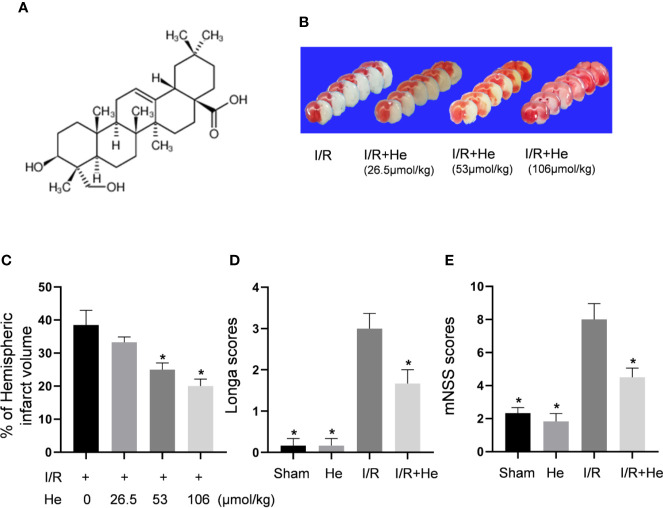
HE reduced infarct volumes and improved neurological deficits after CI/R injury. The mice were injected with HE (different doses) or vehicle (i.p.) once daily for 3 consecutive days after MCAO. **(A)** The molecular structure of HE is shown. **(B)** Mice were injected with HE at doses of 26.5,53 and 106 μmol/kg (i.p.) after MCAO. The representative TTC-stained coronal sections in vehicle- and HE-treated mice are shown. **(C)** HE significantly reduced infarct volumes after CI/R injury. HE treatments significantly reduced Longa **(D)** and mNSS **(E)** scores after CI/R injury. The bar graphs represent the mean ± SEM of 5‑6 brains in each group. *P < 0.05 versus the CI/R group.

The molecular structure of HE, (3beta,4alpha)-3,23-dihydroxyolean-12-en-28-oic acid, is shown in **Figure 2A**. HE treatment decreased the cerebral infarct volume in a dose-dependent manner (P<0.05, **Figure 2B**). HE post-treatment at 26.5umol/kg did not show any significant protective effects compared to the CI/R group (P>0.05, **Figure 2C**); however, HE treatment at higher doses of 53 and 106 μmol/kg provided significant neuroprotection after CI/R injury (P<0.05, **Figure 2C**). Because the 106umol/kg dose produced the most significant effects, this dose was selected for future experiments.

While the CI/R group exhibited poor neurological function by the Longa and mNSS scores, HE treatment (106 μmol/kg, i.p.) significantly alleviated the neurological deficits of the mice. Compared to the CI/R group, Longa and mNSS scores were significantly lower in the HE treatment group (P<0.05, **Figures 2D, E**), indicating that HE treatment provided protective effects against CI/R injury in mice.

### HE Suppressed the Apoptosis of Nerve Cells After CI/R Injury

**Figure 3:**
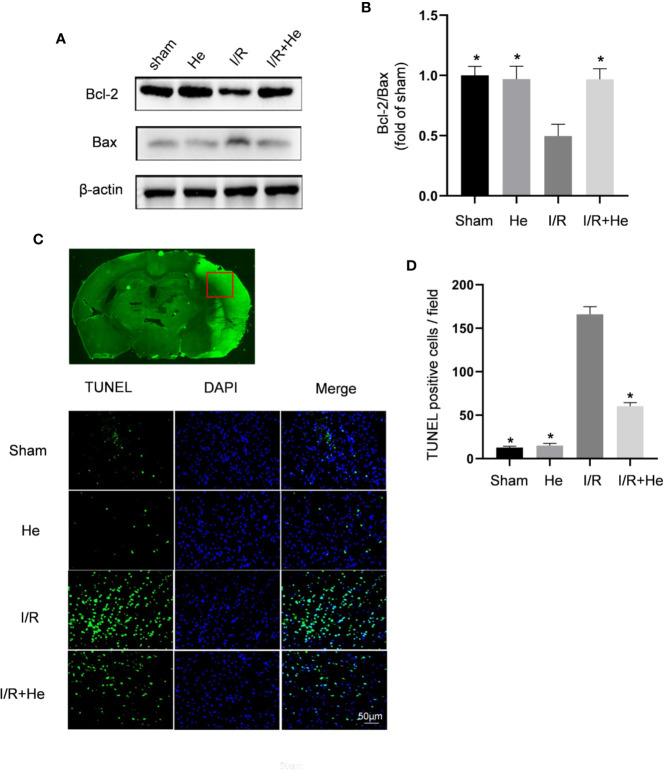
HE suppressed the apoptosis of neurons after CI/R injury. Mice were treated with HE (106 μmol/kg) or vehicle (i.p.) once daily for 3 consecutive days after MCAO. Western blot was used to detect the expression levels of apoptosis-related proteins, and TUNEL staining was used to measure the apoptotic levels after CI/R injury. **(A)** Representative images show the expression levels of Bcl-2, Bax by Western Blot analysis with β-actin as a loading control. **(B)** Quantitative analysis of the Bcl-2 to Bax ratio. **(C)** TUNEL-positive cells showed green fluorescence, and DAPI-positive cells showed blue fluorescence. **(D)** Quantification of the number of TUNEL-positive neural cells in each group. HE treatment significantly decreased the number of TUNEL-positive neural cells after CI/R injury. Scale bar, 25 µm; magnification, 20x. The bar graphs represent the mean ± SEM of three brains in each group. *P < 0.05 versus the CI/R group.

Inhibiting the apoptotic effects of neurons in the ischaemic penumbra is considered a key target for protection against CI/R injury (Chen et al., 2017; Zhang et al., 2018). Previous studies have shown that the change in Bcl-2/Bax ratio reflects the level of apoptosis (Chen et al., 2017). In this study, Western blot analysis of Bcl-2 and Bax was performed on brain tissue in mice after MCAO and 3 days of reperfusion. Consistent with the previous results, the expression ratio of Bcl-2/Bax was decreased in the CI/R group compared with those in the sham and HE groups. However, HE treatment resulted in an increase in Bcl2/Bax ratio compared to the CI/R group (P <0.05, **Figures 3A, B**) with increased Bcl-2 expression and decreased Bax expression. TUNEL staining has also been used as an apoptotic marker (Xu et al., 2019; Zheng et al., 2019). The number of TUNEL-positive cells in brain tissue was significantly decreased after HE treatment compared to the CI/R group (P < 0.05, **Figures 3C, D**). These results further support the anti-apoptotic effects of HE in CI/R injury.

### HE Alleviated the Inflammatory Response After CI/R Injury

**Figure 4:**
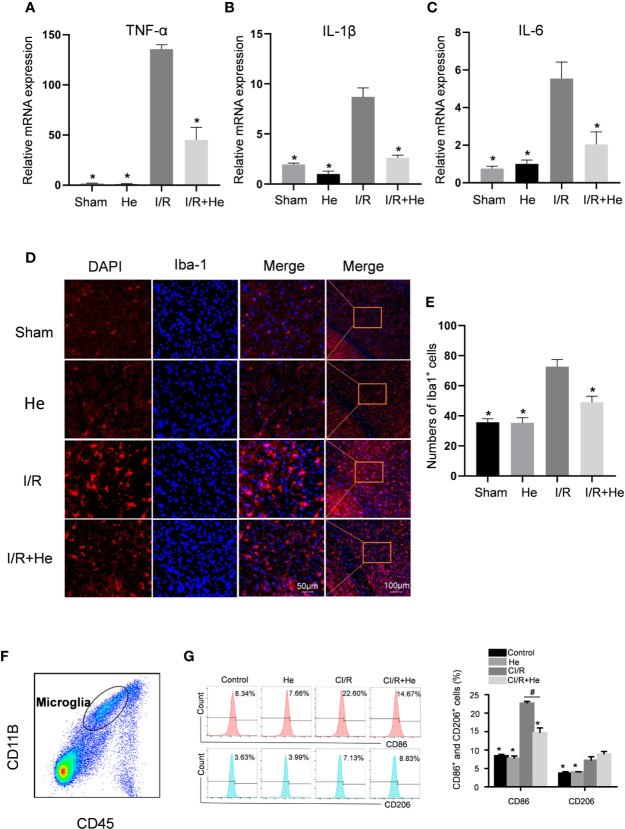
HE alleviated the inflammatory response after CI/R injury. The mice were treated with HE (106 μmol/kg, i.p.) or vehicle once daily for 3 consecutive days after CI/R injury. Pro-inflammatory cytokine levels were determined by qRT-PCR. Immunofluorescence staining was used to detect the number of Iba-1-positive microglia in the ischaemic penumbra. **(A–C)** The mRNA levels of TNFα, IL-1β, and IL-6 in the ischaemic penumbra tissues. HE significantly decreased the mRNA levels of TNFα, IL-1β, and IL-6 after CI/R injury. **(D)** Immunofluorescence staining of Iba-1-positive microglia in the ischaemic penumbra area. Iba-1 is shown as red fluorescence, and DAPI is shown as blue fluorescence. **(E)** Quantitative analysis of the number of Iba-1-positive microglia in the cortex ischaemic penumbra area. **(F)** Purity of microglia (CD45^mid^CD11b+) was measured by flow cytometry. **(G)** The percentages of CD11b^+^CD45^mid^CD86^+^ and CD11b^+^CD45^mid^CD206^+^ microglia were analysed by flow cytometry. The bar graphs represent the mean ± SEM of three brains in each group. *P < 0.05 versus the CI/R group. ^#^P < 0.05 versus the CI/R+He group.

Inflammation has been demonstrated to be involved in all stages of CI/R injury. To explore the potential anti-inflammatory effects of HE, we detected the mRNA expression levels of pro-inflammatory cytokines, including TNFα, IL-1β and IL-6, by quantitative real-time polymerase chain reaction (qPCR). The mRNA levels of TNFα, IL-1β, and IL-6 were all decreased after HE treatment compared with the CI/R group (P<0.05, **Figures 4A–C**). However, HE treatment did not influence the mRNA levels of anti-inflammatory cytokines IL-4 and IL-10 compared with the CI/R group (P>0.05, **Supplementary Figures 1C, D**).

The activation level of microglia is considered to be a marker of the severity of inflammation after CI/R injury (Li et al., 2019) and this was quantified by Iba-1 immunofluorescence staining. A key characteristic of activated microglia following CI/R injury are their amoeba-like cell bodies (**Figure 3**). The number of Iba-1-positive microglia was significantly increased in infarcted tissue compared with the sham and HE groups. Intriguingly, HE treatment decreased the number of Iba-1-positive microglia after CI/R injury (**Figures 4D, E**). To further investigate the role of HE in microglia, flow cytometry was used to determine the levels of M1-pro-inflammatory and M2-anti-inflammatory microglia on day 3 after CI/R injury. CD11b^+^CD45^mid^CD86^+^ was used to label M1-pro-inflammatory microglia, and CD11b^+^CD45^mid^CD206^+^ was used to label M2-anti-inflammatory microglia. Our results show that the two phenotypes of activated microglia were both upregulated after CI/R injury. Interestingly, HE significantly reduced the percentage of only the M1-pro-inflammatory microglia, but had no effects on the percentage of M2-anti-inflammatory microglia (P<0.05, **Figures 4F, G**). These results indicate that HE significantly alleviated the inflammatory response after CI/R injury.

### HE Inhibited the NF-kB p65 Signalling Pathway After CI/R Injury

**Figure 5:**
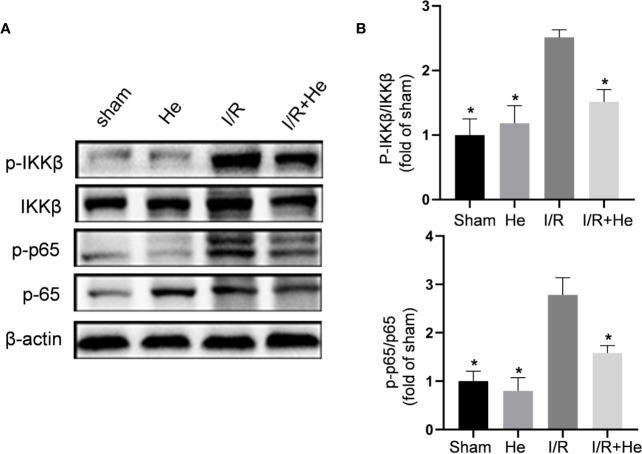
HE inhibited the NF-kB p65 signalling pathway after CI/R injury. The mice were treated with HE (106 μmol/kg, i.p.) or vehicle once daily for 3 consecutive days after ischaemia. The protein levels were detected by Western blot. **(A)** The representative images show the levels of p-IKKβ, IKKβ, p-p65, p65, and β-tubulin by Western blotting. β-actin was used as a loading control. **(B)** Quantitative analysis showed that HE decreased the expression levels of p-IKKβ and p-p65 after cerebral CI/R injury. The bar graphs represent the mean ± SEM of three brains in each group. *P < 0.05 versus the CI/R group.

Nuclear factor-kappa B (NF-kB) plays a central role in the pro-inflammatory function through the regulation of inflammatory cytokines after CI/R injury (Li et al., 2019). HE has been reported to inactivate lipopolysaccharide-stimulated RAW 264.7 cells by inhibiting the NF-kB signalling pathway (Lee et al., 2015). In our study, the core proteins of the NF-kB signalling pathway were detected by Western blot. CI/R injury significantly increased the levels of phospho-IKKβ and phospho-p65 compared with the sham and HE groups. However, HE treatment downregulated phospho-IKKβ, phospho-IκBα and phospho-p65 levels compared with the CI/R group (**Figure 5**). Taken together, these data indicated that HE inhibits the NF-kB signalling pathway during CI/R injury.

### HE Alleviated CI/R Injury Through Inhibiting the MEK4/7-JNK Axis

**Figure 6:**
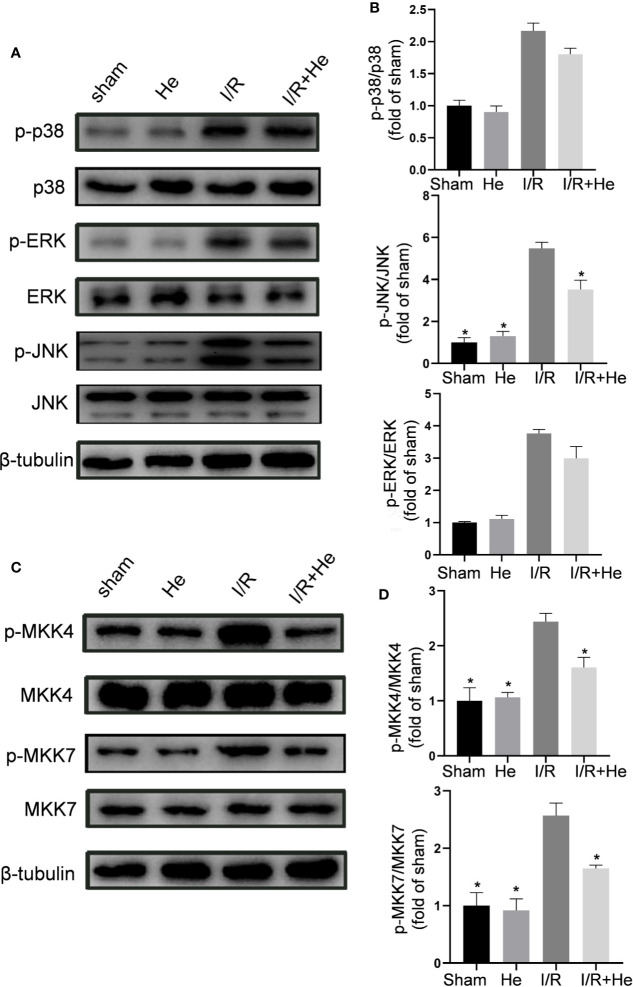
HE alleviated CI/R injury through inhibiting the MEK4/7/JNK axis. Mice were treated with HE (106 μmol/kg, i.p.) or vehicle once daily for 3 consecutive days after ischaemia. After 60 min of ischaemia and 3 days of reperfusion, cerebral tissue was collected, and protein levels were detected by Western blot. **(A)** The representative images show the levels of p-p38, p38, p-ERK, ERK, p-JNK, JNK, and β-tubulin. β-tubulin was used as a loading control. **(B)** Quantitative analysis showed that HE decreased the expression level of p-JNK, but not that of p-ERK or p-p38, after CI/R injury. **(C)** Representative images show the levels of p-MKK4, MKK4, p-MKK7, MKK7, and β-tubulin. β-tubulin was used as a loading control. **(D)** Quantitative analysis showed that HE decreased the expression levels of p-MKK4 and p-MKK7 after CI/R injury. The bar graphs represent the mean ± SEM of three brains in each group. *P < 0.05 versus the CI/R group.

Following CI/R injury, activation of the MAPK pathways P38, ERK, and JNK induces the production of pro-inflammatory cytokines/chemokines and cellular apoptosis (Yang et al., 2019). HE has shown its capacity to suppress the MAPK pathway in other disease models. To determine whether HE protected against CI/R injury through MAPK pathway inactivation, levels of p-p38, p-ERK, and p-JNK were assayed by Western blot analysis (**Figure 6A**). Interestingly, HE treatment did not affect the levels of p-p38 or p-ERK after CI/R injury (P>0.05, **Figure 6B**) but decreased the level of p-JNK compared with the CI/R group (P<0.05, **Figure 6B**). To further elucidate the molecular mechanism of HE action, we measured the expression levels of MKK4 and MKK7, the upstream proteins of JNK (**Figure 6C**). Quantitative analysis confirmed the differences in the ratios of p-MKK4 to MKK4 between the CI/R group and the HE group. The levels of p-MKK4 to MKK4 and p-MKK7 to MKK7 in the HE treatment group were significantly decreased compared with those in the CI/R group (P<0.05, **Figure 6D**). These results indicated that HE may protect against CI/R injury through inhibiting the MKK4/7-JNK axis.

### HE Protected Against CI/R Injury in an MLK3-Dependent Manner

**Figure 7:**
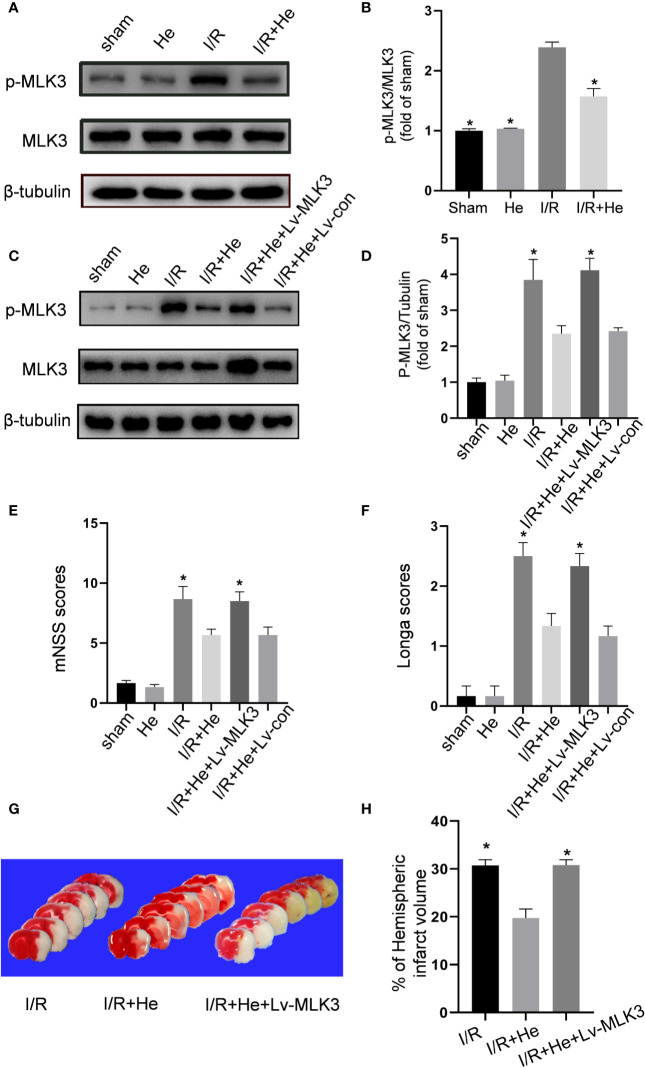
HE protected against CI/R injury in an MLK3-dependent manner. The mice were treated with HE (106 μmol/kg, i.p.) or vehicle once daily for 3 consecutive days after ischaemia. After 60 min of ischaemia and 3 days of reperfusion, cerebral tissue was collected, and protein levels were detected by Western blot. **(A)** Representative images show the expression levels of p-MLK3, MLK3 and β-tubulin. β-tubulin was used as a loading control. **(B)** Quantitative analysis shows that HE decreased the expression of p-MLK3 after CI/R injury. The bar graphs represent the mean ± SEM of three brains in each group. *P < 0.05 versus the CI/R group. **(C)** Lentiviral vectors overexpressing MLK3 (Lv-MLK3) or control (Lv-con) were injected using a stereotactic instrument into the cortex of mice 14 days before MCAO. After reperfusion, HE (106umol/kg, i.p.) was administered once daily for 3 consecutive days. Representative images show the levels of p-MLK3, MLK3, and β-tubulin. β-tubulin was used as a loading control. **(D)** Quantitative analysis showed that Lv-MLK3, and not Lv-con, increased the expression levels of p-MLK3 and MLK3 after HE treatment after CI/R injury. **(E, F)** The Longa and mNSS scores were evaluated after CI/R injury. Activating MLK3 almost reversed the protective effects of HE on saving the neurological deficits. **(G)** Representative TTC-stained coronal sections in each group are shown. **(H)** Statistical analysis of infarct volumes was performed in each group. Activating MLK3 almost reversed the protective effects of HE on decreasing the infarct volumes after CI/R injury. The bar graphs represent the mean ± SEM of 5‑8 brains in each group. *P < 0.05 versus the CI/R+HE group.

Since HE inhibited both the NFκB and MAPK signals during CI/R injury, we hypothesized that HE treatment may exert its action upon a common regulator of NFκB and MAPK. MLK3 regulates both NFκB and MAPK signals in different pathologic conditions. The effect of HE treatment on phospho-MLK3 expression was then assayed. The levels of p-MLK3 were signifificantly decreased with HE treatment compared to CI/R group (P<0.05, **Figures 7A, B**). Lentiviral vectors overexpressing MLK3 (Lv-MLK3) or a negative control (Lv-con) were transfected to the cerebral cortex of mice for 2 weeks and confirmed by Western blot analysis. The levels of MLK3 and p-MLK3 were significantly increased with HE treatment compared to Lv-con treatment (P<0.05, **Figures 7C, D**). The Longa and mNSS scores in the CI/R+HE+LV group were significantly higher than those in the CI/R + HE group (P<0.05, **Figures 7E, F**). As expected, TTC staining showed that LV-MLK3 abolished the neuroprotective effects of HE treatment in reducing the infarct volumes after CI/R injury (**Figure 7G**). Additionally, the quantitative analysis of infarct volumes confirmed that activated MLK3 signals reversed the neuroprotective effects of HE (P<0.05, **Figure 7H**). Taken together, these results demonstrate that the neuroprotective effects of HE in CI/R injury are through its modulation of the MLK3 signalling pathway.

## Discussion

**Figure 8:**
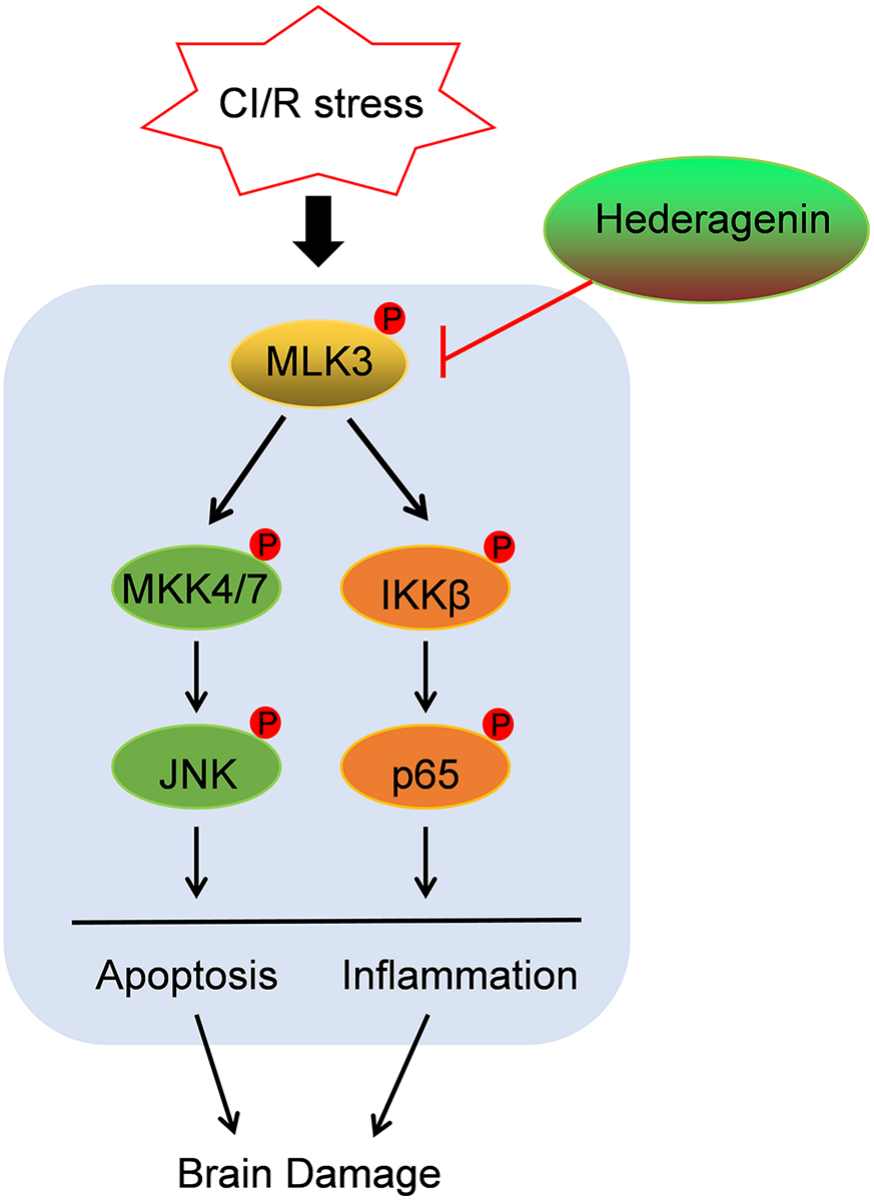
Schematic diagram illustrating the mechanisms of HE treatment-related protective effects against CI/R injury. HE inhibited the phosphorylation of MLK3, which activates the NF-kB and MEK4/7-JNK pathways, leading to inflammation and apoptosis after CI/R injury.

The identification of effective neuroprotective agents remains an important challenge in the treatment of ischaemic stroke. In this study, we demonstrate that the natural plant extract HE provides neuroprotective effects against CI/R injury by improving neurobehavioral function and decreasing brain infarct volumes. HE treatment mitigated both apoptosis and inflammation within the infarcted tissues in CI/R injury. Furthermore, our results suggest the potential mechanism of HE’s neuroprotective function may be through the inhibition of MLK3 phosphorylation, which activates the NF-kB and MEK4/7-JNK pathways (**Figure 8**).

The clinical strategy for ischaemia stroke is plagued by the lack of effective neuroprotectants (Ren et al., 2018). Intravenous thrombolysis is limited due to its short window of opportunity for application, and many drugs have failed in clinical trials due to their inefficacy and toxicity (Yu et al., 2019). Therefore, finding new neuroprotectants is crucial for CI/R injury treatment. Herbal medicine is widely used for ischaemic stroke in China. Their safety and efficacy have been confirmed in clinical applications (Feng et al., 2019); however, it has been difficult to identify the effective constituents and active ingredients within these herbal compounds (Prendergast et al., 2017).

HE has been reported to have many pharmacological effects, including anti-platelet aggregation, anti-inflammatory, and anti-apoptotic effects (Lee et al., 2015; Lu et al., 2015). Pharmacological research has demonstrated that HE is capable of penetrating the blood brain barrier (BBB) (Yang et al., 2011). HE decreases the expression of the mRNA for the serotonin transporter in the treatment of depression and has also been shown to have a therapeutic roles in neurodegenerative disorders through the promotion of autophagy of neurodegenerative mutant disease proteins (Liang et al., 2015; Wu et al., 2017).

In this study, mice underwent peritoneal HE injections after the induction of the MCAO model for 3 days. Our results show that HE improves the neurological behavior function and decreases the infarct volumes of mice after CI/R injury. Apoptosis and inflammation are the main pathological mechanisms of CI/R injury (Su et al., 2017; Li et al., 2018). Local ischaemia first leads to neuronal apoptosis (Zhang C. Y. et al., 2019). Then, reperfusion injury leads to an inflammatory storm, which further potentiates cellular apoptosis (Wu et al., 2019). HE has been demonstrated to have anti-apoptotic and anti-inflammatory effects in other disease processes (Kim et al., 2017; Ma et al., 2020). In our study, HE attenuated the number of apoptotic cells and increased the ratio of Bcl-2/BAX. Although HE significantly attenuated the transcription of pro-inflammatory cytokines, there was no demonstrated effect on the transcription of anti-inflammatory cytokines. After CI/R injury, the newly activated microglia switch to either the M1-pro-inflammatory or the M2-anti-inflammatory phenotype (Guruswamy and ElAli, 2017). The level and phenotypes of activated microglia are closely related to neuroinflammation in the infarcted areas (Takada et al., 2020). In this study, HE treatment significantly decreased the number of IBA1-positive cells within brain tissue. Concurrently, HE decreased the percentage of M1-pro-inflammatory microglia after CI/R injury. The above results demonstrate that HE exerts its neuroprotective effects by reducing apoptosis and inflammatory responses after CI/R injury.

The protective mechanisms of HE in different diseases are still unclear. Gyeong-Ji Kim et al. show that HE alleviates the pro-inflammatory and apoptotic pathways by inhibiting p38MAPK signalling in a rat model of alcohol intoxication (Kim et al., 2017). In addition, HE decreased the production of intracellular reactive oxygen species in ovariectomy-induced bone loss through the inhibition of p38 and Erk signalling. Although there is no clearly defined mechanism for HE on ischaemia/reperfusion injury, other studies have implicated the involvement of the MAPK pathway (Li et al., 2017; Xie et al., 2018). Intriguingly, HE induced the activation of JNK, but not ERK or p38. Furthermore, HE decreased the activation of MKK4/7, suggesting that the MKK4/7-JNK axis is potential involved in HE’s protective function. Lu SH et al. demonstrated the has anti-atherosclerotic effects of HE through the inhibition of the NFκB (Lu et al., 2015). As the MAPK and NFκB pathways were both suppressed by HE treatment, we hypothesized that there may be a common mechanism for the protective effects of HE during CI/R injury. MLK3 is a member of the serine/threonine kinase family and activates JNK signalling through the phosphorylation of IKK-a and IKK-b (Ng et al., 2001). Substantial evidence has demonstrated that MLK3 regulates MAPK and NFκB pathway activity after CI/R injury (Hu et al., 2012; Sun et al., 2019). Anemarsaponin B, another steroid saponin, is a specific blocker of MLK3 signalling in inflammatory conditions (Kim et al., 2009). Therefore, we speculated that HE may be also be a potential inhibitor of MLK3 signalling. Consistent with previous results, HE downregulated MLK3 activity during CI/R injury. Furthermore, after overexpression of MLK3 on ischaemic hemispheres abrogated HE’s protective effects in the MCAO mouse model. Our results demonstrated that HE treatment decreased the activation of MLK3 and upregulated the MAPK and NFκB pathways; however, the exact mechanism of how HE regulates MLK3 requires further exploration.

In summary, HE provides neuroprotection against apoptosis and inflammation in an MCAO mouse model. HE treatment decreased the activation of the MLK3 signalling pathway, which potentiates CI/R damage *via* the MAPK and NFκB pathways. One limitation of our study is that the chosen test concentration of HE (106 μmol/kg,i.p.), may be too high to achieve in human translational trials (Heinrich et al., 2020). Although, the lower dose of 53 μmol/kg i.p also demonstrated significant neuroprotection after CI/R injury and should be further investigated. Future research including the utilization of nano-formulations of HE into liposomes will allow for more targeted delivery of HE. This may allow for more soluble, stable, and bioavailable drug delivery in the treatment of ischemic strokes (Coimbra et al., 2012).

## Data Availability Statement

The raw data supporting the conclusions of this article will be made available by the authors, without undue reservation.

## Ethics Statement

The animal study was reviewed and approved by Animal Ethics Committee of Nanjing University.

## Author Contributions

Conceived and designed the experiments: YX, WY, and HY. Performed the experiments: HY, WL, XC, JC, YZ, and LS. Analyzed the data: LS, HY, and JL. Contributed reagents/materials/analysis tools: XC and YC. Contributed to the writing of the manuscript: HY, LS, and XC.

## Funding

This research was funded by the National Natural Science Foundation of China (81920108017, 81630028, 81701170, 81400963), Jiangsu Province Key Medical Discipline (ZDXKA2016020) and Jiangsu Provincial Medical Youth Talent (Grant #QNRC2016327 and #QNRC2016328).Yangzhou 13th Five-Year Science and Education Key Talent Fund (ZDRC54, ZDRC55). The Young Talent Support Program from Jiangsu Association for Science and Technology.

## Conflict of Interest

The authors declare that the research was conducted in the absence of any commercial or financial relationships that could be construed as a potential conflict of interest.

## Supplementary Material

Supplement Figure 1 **(A)** Regional CBF Doppler imaging was monitored using two-dimensional laser speckle imaging techniques 15 min before MCAO, 15 min after the onset of MCAO. **(B)** A Lv-MLK3 lentiviral vector expressing GFP was infected in the mouse brain cortex using a stereotactic instrument. **(C, D)** The mRNA levels of IL-4 and IL-10 in the ischaemic penumbra tissues. There was no significant difference in the CI/R group compared with the CI/R+HE group.
