# Supplementary figures and images for: Hederagenin Attenuates Cerebral Ischaemia/Reperfusion Injury by Regulating MLK3 Signalling
Source: Front Pharmacol. 2020 Jul 30;11:1173. doi: 10.3389/fphar.2020.01173 (PMC7406912; doi:10.3389/fphar.2020.01173)

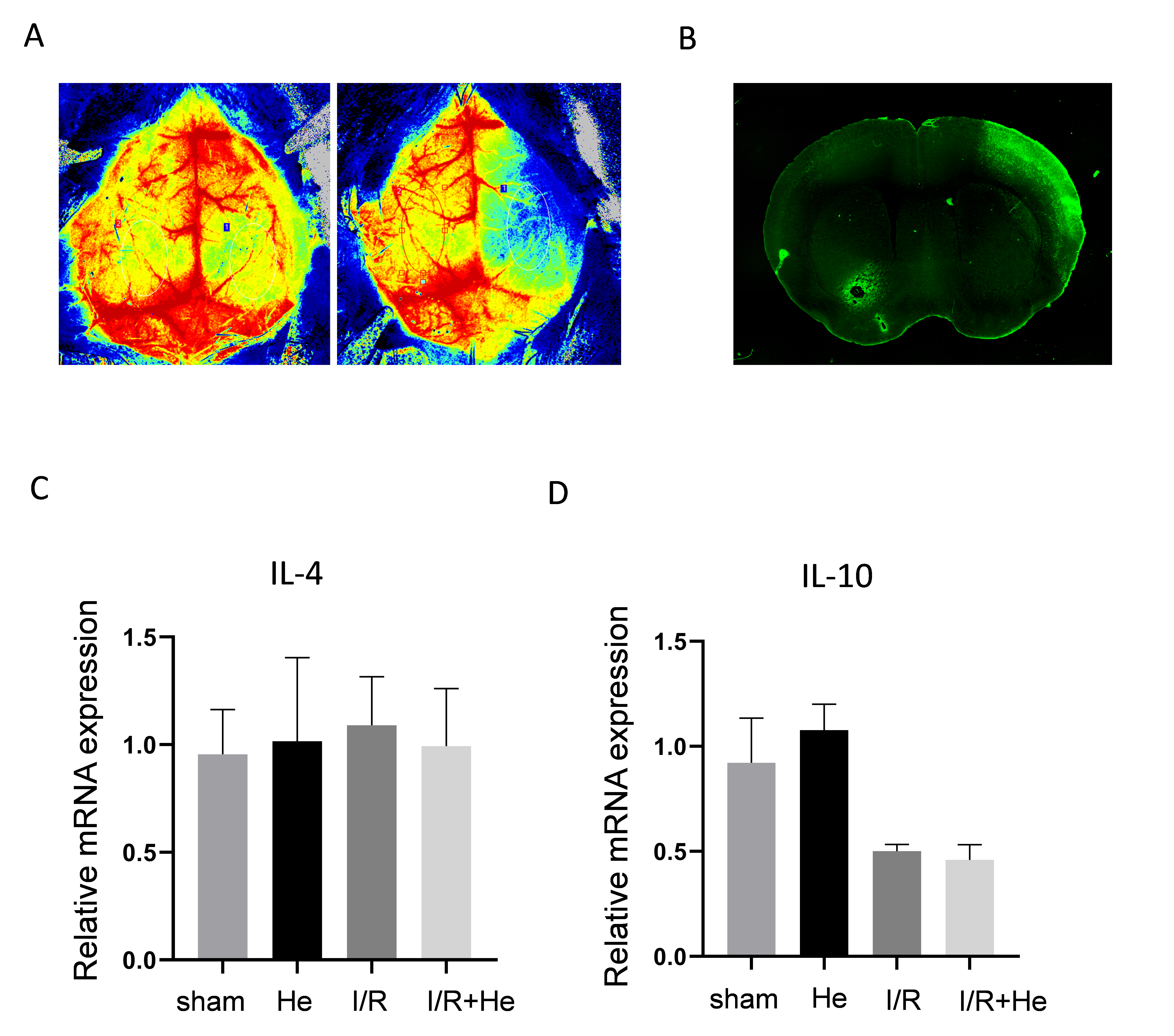

Supplement: Supplement Figure 1 — (A) Regional CBF Doppler imaging was monitored using two-dimensional laser speckle imaging techniques 15 min before MCAO, 15 min after the onset of MCAO. (B) A Lv-MLK3 lentiviral vector expressing GFP was infected in the mouse brain cortex using a stereotactic instrument. (C, D) The mRNA levels of IL-4 and IL-10 in the ischaemic penumbra tissues. There was no significant difference in the CI/R group compared with the CI/R+HE group. [file Image_1.tif]
